# Identification of cucurbit chlorotic yellows virus P4.9 as a possible movement protein

**DOI:** 10.1186/s12985-019-1192-y

**Published:** 2019-06-20

**Authors:** Ying Wei, Yajuan Shi, Xaioyu Han, Siyu Chen, Honglian Li, Linlin Chen, Bingjian Sun, Yan Shi

**Affiliations:** College of Plant Protection, Henan Agricultural University, Zhengzhou, 450002 China

**Keywords:** CCYV, P4.9, Movement

## Abstract

**Background:**

Cucurbit chlorotic yellows virus (CCYV) is a bipartite cucurbit-infecting crinivirus within the family *Closteroviridae*. The crinivirus genome varies among genera. P4.9 is the first protein encoded by CCYV RNA2. P5, which is encoded by LIYV, is necessary for efficient viral infectivity in plants; however, it remains unknown whether CCYV P4.9 is involved in movement.

**Finding:**

In this study, we used green fluorescent protein (GFP) to examine the intracellular distribution of P4.9-GFP in plant cells, and observed fluorescence in the cytoplasm and nucleus. Transient expression of P4.9 was localized to the plasmodesmata. Co-infiltration of agrobacterium carrying binary plasmids of P4.9 and GFP facilitated GFP diffusion between cells. Besides P4.9 was able to spread by itself to neighboring cells, and co-localized with a marker specific to the endoplasmic reticulum, HDEL-mCherry, but not with the Golgi marker Man49-mCherry.

**Conclusions:**

Together, these results demonstrate that CCYV P4.9 is involved in cell–cell movement.

**Electronic supplementary material:**

The online version of this article (10.1186/s12985-019-1192-y) contains supplementary material, which is available to authorized users.

## Main text

Plant viruses are small obligate intracellular parasites that live in the symplast of their hosts. Plant virus movement is an essential component of complete viral infection. Cell–cell movement, through the cell walls separating adjacent cells, involves movement proteins (MPs) that alter the size exclusion limit (SEL) of the plasmodesmata (PD), enabling transport of the viral genome into adjacent cells [1, 2].

Cucurbit chlorotic yellows virus (CCYV) is a cucurbit-infecting crinivirus within the family *Closteroviridae* [3–5]. Members of this family possess the largest, most complex single-stranded RNA (ssRNA) genomes, which vary in size from ca. 15 to 20 kb [6]. Like most members of the *Crinivirus* genus, CCYV has a bipartite genome. The crinivirus genome varies among species in the RNA1 3′ region downstream of the RNA-dependent RNA polymerase, where the number of open reading frames (ORFs) varies from zero to three. CCYV RNA2 contains eight ORFs, which encode P4.9, HSP70h, P6.5, P59, P9, CP (major coat protein), CPm (minor coat protein), and P26. P4.9, HSP70h, P59, CP, and CPm are quintuple gene blocks that are conserved among all members of the family *Closteroviridae* [7].

Beet yellows virus (BYV) is a well-studied closterovirus that requires five proteins for cell–cell movement: P6, HSP70h, P64, CP, and CPm [8–10]. Similarly, Citrus tristeza virus (CTV) proteins involved in viral assembly also have functions in viral spread; these include HSP70h, P61, CP, and minor CP (CPm). Two other proteins, P6 and P33, are not required for assembly but are involved in virus movement [11, 12]. A non-virion protein, P26, which is encoded by lettuce infectious yellows crinivirus, is PD-localized and essential for viral systemic infection [13]. Small proteins P5 and P9, which are encoded by LIYV RNA2, are indispensable for efficient viral infectivity in plants [14]. Movement proteins differ among cloteroviruses; therefore, the objective of this study was to determine whether the CCYV protein P4.9 is a movement protein.

**Fig. 1:**
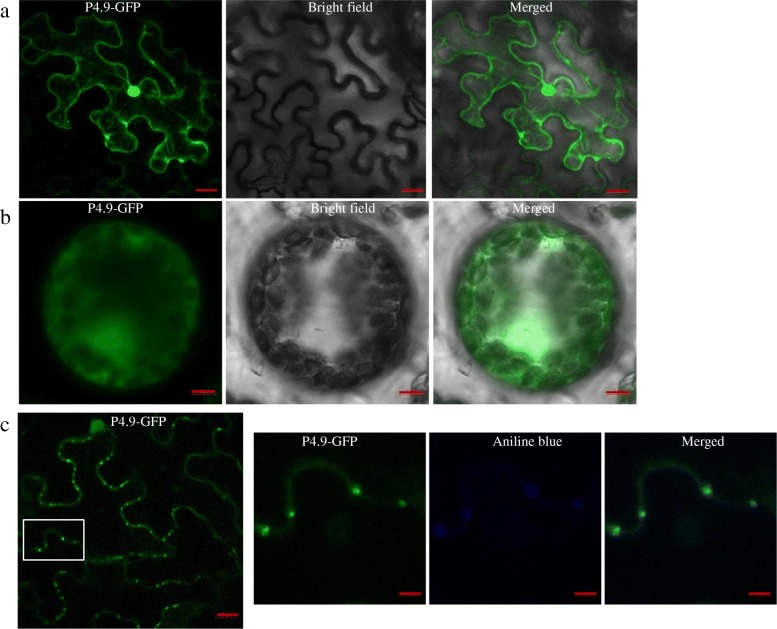
(**a**) Subcellular localization of P4.9-GFP, constructed using green fluorescent protein (GFP), was observed in *Nicotiana benthamiana* leaves at 2 days post-inoculation (dpi). Bar, 20 μm. **b** Distribution of P4.9-GFP in protoplasts isolated from agrobacterium-infiltrated *N. benthamiana* leaves at 2 dpi. Bar, 5 μm. **c** P4.9-GFP accumulated in punctate spots along the cell periphery, co-localized with the plasmodesmata marker dye aniline blue at 2 dpi. Bars, 20 μm (left) and 5 μm (right)

Based on an amino acid sequence analysis performed using the PSORT online tool, we hypothesized that the localization of P4.9 is extracellular, including the cell wall. To determine the intracellular localization of P4.9, we cloned the protein by fusion to the C-terminus of green fluorescent protein (GFP). To construct P4.9-GFP, we amplified the polymerase chain reaction (PCR) product using the primer pair BPp4.9F/BPp4.9R and BDp4.9 as template (Additional file 1: Table S1), gateway technology, and the pEarlyGate 103 vector [15]. The resulting clone was transformed into *Agrobacterium tumefaciens* strain GV3101 for agroinfiltration. P4.9-GFP was visualized using laser scanning confocal microscopy (LSCM) at 2 days post-inoculation (dpi). GFP fluorescence was observed in the cytoplasm and nucleus (Fig. 1a).

To further characterize the intracellular localization pattern, we treated *Nicotiana benthamiana* leaves expressing P4.9-GFP with a cell wall degrading enzyme to obtain protoplast, as previously described [11]; localization within the protoplast was consistent with our microscopy results (Fig. 1b). The fluorescing tubules protruding from the cell surface was not found as decribed for *Citrus tristeza virus* (CTV) p33 by Bak (2015). To determine the PD localization of P4.9, we used the callose-binding plasmodesmata marker dye aniline blue. We agrofiltrated 0.1 mg/mL aniline blue into leaves for 2 h, and then detached the leaves for fluorescence observation. Fluorescence signals were individually visualized using LSCM; we found that P4.9 accumulated in punctate spots along the cell periphery (Fig. 1c), which is characteristic of PD localization. We then demonstrated co-localization of these sites using aniline blue, confirming that P4.9 is PD-localized (Fig. 1c).

**Fig. 2:**
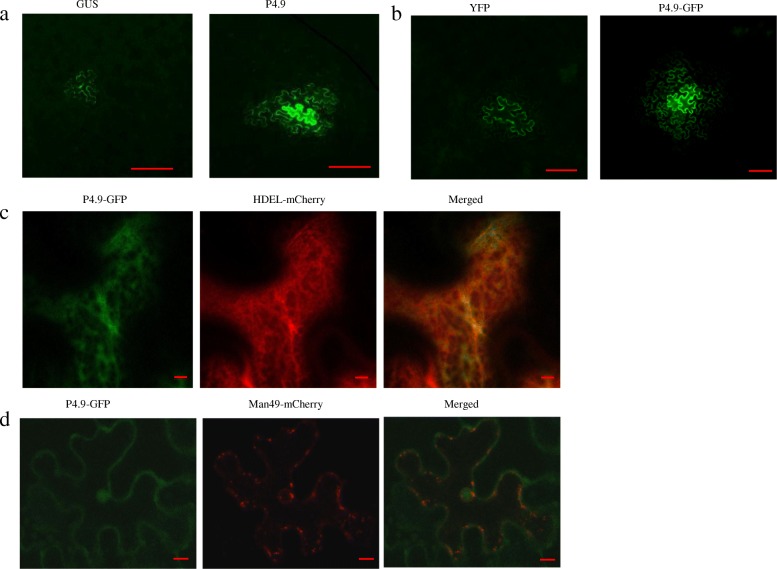
Functional analysis of P4.9 in cell–cell movement. **a** P4.9 facilitated diffusion of GFP in *N. benthamiana* leaf epidermis. Agrobacterium carrying plasmid of GFP was diluted 1000-fold. Bar, 200 μm. **b** Cell-to-cell spread of P4.9-GFP in the absence of other viral components. Agrobacterium carrying plasmid of P4.9-GFP and YFP was diluted 4000-fold. Bar, 100 μm. **c** Co-localization of P4.9-GFP with the ER marker HDEL-mCherry in *N. benthamiana* leaf epidermis. Bar, 5 μm. **d** Co-expression of P4.9-GFP with the Golgi apparatus marker Man49-mCherry in *N. benthamiana* leaf epidermis. Bar, 10 μm

A distinguishing property of MPs is the ability to increase the SEL and enhance cell-to-cell trafficking. To construct a P4.9 binary expression vector, we amplified its PCR product using primer pair pGDP4.9PstIF/pGDP4.9BamHIR (Additional file 1: Table S1), digested it using *Pst*I and *Bam*HI, and cloned it into the pGDMyc vector to generate pGDP4.9. Agrobacteria carrying the GFP binary plasmid was diluted 1000-fold, mixed with agrobacterium cultures carrying binary plasmid of P4.9 or GUS, and co-infiltrated into *N. benthamiana* leaves. Leaf samples were viewed for GFP fluorescence 2 days post-infiltration using a fluorescence microscope (ECLIPSE Ti-S, Nikon) at 10× magnification. GFP fluorescence was mainly restricted to single cells when co-expressed with GUS. P4.9 promoted the diffusion of GFP fluorescence (Fig. 2a). We then estimated the number of cells expressing GFP. Compared with GUS control, P4.9 co-expression with GFP promoted GFP movement. Significantly more cell clusters expressed GFP in 2–3 cells and > 4 cells than in the control (Table 1). To further measure the intercellular capacity of P4.9, agrobacterium carrying P4.9-GFP or YFP control was diluted 4000-fold. YFP was retained in individual epidermal cells, whereas P4.9-GFP spread to neighboring cells (Fig. 2b).

**Table 1:** P4.9 promoted cell–cell movement of free GFP in *Nicotiana benthamiana.* Asterisks denote significant differences compared with the results of GUS (*P*<0.05)

	1 cells	2–3 cells	4–5 cells	Total foci counted
P4.9	148 (56.7%)*	103 (39.5%)*	10 (3.8%)*	261
GUS	93 (78.2%)	25 (21%)	1 (0.8%)	119

The endoplasmic reticulum (ER) membrane has been reported to play essential roles in MP and virus cell–cell movement by diffusing between cells through the PD [16, 17]. Therefore, P4.9-GFP was co-expressed with the ER marker HDEL-mCherry in *N. benthamiana* leaves; P4.9 was co-localized with the ER marker (Fig. 2c). We also examined the localization of P4.9 in the Golgi apparatus using the marker Man49-mCherry (Arabidopsis Biological Resource Center, ABRC). LSCM showed that P4.9-GFP did not co-localize with the Golgi apparatus (Fig. 2d), indicating that intracellular trafficking of P4.9 is independent of the ER–Golgi secretory pathway.

Different plant viruses use different host cell transport machinery to move from one cell to another through the PD [17–21]. A previous study of LIYV P5 and P9 showed that neither protein was essential for viral infection, but that knocking out either protein led to debilitated LIYV infection [14], suggesting that these two proteins may participate in viral movement. Similarly, the P6 protein of closteroviruses BYV and CTV has been found to be involved in viral movement [10–12].

In this study, we found that transient expression of P4.9 was localized to the PD, and facilitated GFP diffusion between cells. P4.9 was co-localized with the ER-specific marker ER-Cherry but not the Golgi marker Manl-Cherry. Our results provide direct evidence that the CCYV protein P4.9 is involved in cell–cell movement. To our knowledge, this is the first such demonstration for a member of the genus *Crinivirus*.

## Additional file

Additional file 1: **Table S1**. Primers used in this study. Restriction sites were underlined, and start and stop codons were shown in italics. The English in this document has been checked by at least two professional editors, both native speakers of English. For a certificate, please see http://www.textcheck.com/certificate/JDS64O. (PDF 400 kb)

## Data Availability

Not applicable.

## Abbreviations

CCYV: Cucurbit chlorotic yellows virus
GFP: Green fluorescent protein
MP: Movement proteins
PD: Plasmodesmata
SEL: Size exclusion limit
