# Supplementary material for: Identification of cucurbit chlorotic yellows virus P4.9 as a possible movement protein
Source: Virol J. 2019 Jun 20;16:82. doi: 10.1186/s12985-019-1192-y (PMC6587283; doi:10.1186/s12985-019-1192-y)
Supplement: Supplementary file 1 — Table S1. Primers used in this study. Restriction sites were underlined, and start and stop codons were shown in italics. The English in this document has been checked by at least two professional editors, both native speakers of English. For a certificate, please see http://www.textcheck.com/certificate/JDS64O. (PDF 400 kb) [file 12985_2019_1192_MOESM1_ESM.pdf]

Table S1 Primers used in the paper

Primers	Primer Sequences
BPp4.9F	GGGGACAAGTTTGTACAAAAAAGCAGGCTTC*ATG*CCAAAAGCTTTCAAATT
BPp4.9R	GGGGACCACTTTGTACAAGAAAGCTGGGTCCGACAACAGATAGATTGAA
pGDP4.9PstIF	CGACTGCAG*ATG*CCAAAAGCTTTCAAATT
pGDP4.9BamHIR	CGCGGATCC*TTA*CGACAACAGATAGATTGA

带格式表格

删除的内容: P

带格式的：字体：倾斜

删除的内容: BPRNA2P4

带格式的：下划线

带格式的：字体：倾斜

删除的内容: .

带格式的：下划线

带格式的：字体：倾斜

删除的内容: .

删除的内容: .
Table S2 P4.9 promoted cell-to-cell movement of free GFP in *Nicotiana benthamiana* .
...
